# Uncoupling Protein 2: A Key Player and a Potential Therapeutic Target in Vascular Diseases

**DOI:** 10.1155/2017/7348372

**Published:** 2017-10-15

**Authors:** Giorgia Pierelli, Rosita Stanzione, Maurizio Forte, Serena Migliarino, Marika Perelli, Massimo Volpe, Speranza Rubattu

**Affiliations:** ^1^Department of Cardiovascular Disease, Tor Vergata University of Rome, Rome, Italy; ^2^IRCCS Neuromed, Pozzilli, Isernia, Italy; ^3^Department of Clinical and Molecular Medicine, School of Medicine and Psychology, Sapienza University of Rome, Rome, Italy

## Abstract

Uncoupling protein 2 (UCP2) is an inner mitochondrial membrane protein that belongs to the uncoupling protein family and plays an important role in lowering mitochondrial membrane potential and dissipating metabolic energy with prevention of oxidative stress accumulation. In the present article, we will review the evidence that UCP2, as a consequence of its roles within the mitochondria, represents a critical player in the predisposition to vascular disease development in both animal models and in humans, particularly in relation to obesity, diabetes, and hypertension. The deletion of the UCP2 gene contributes to atherosclerosis lesion development in the knockout mice, also showing significantly shorter lifespan. The UCP2 gene downregulation is a key determinant of higher predisposition to renal and cerebrovascular damage in an animal model of spontaneous hypertension and stroke. In contrast, UCP2 overexpression improves both hyperglycemia- and high-salt diet-induced endothelial dysfunction and ameliorates hypertensive target organ damage in SHRSP. Moreover, drugs (fenofibrate and sitagliptin) and several vegetable compounds (extracts from Brassicaceae, berberine, curcumin, and capsaicin) are able to induce UCP2 expression level and to exert beneficial effects on the occurrence of vascular damage. As a consequence, UCP2 becomes an interesting therapeutic target for the treatment of common human vascular diseases.

## 1. Introduction

Uncoupling proteins (UCPs) belong to the family of mitochondrial transporter proteins and are important for lowering mitochondrial membrane potential and dissipating metabolic energy as heat, maintenance of respiration, glucose disposal rate, insulin secretion, and prevention of reactive oxygen species (ROS) accumulation [1, 2].

Five UCPs have been characterized in mammals: UCP1–UCP3 that are evolutionary related and UCP4 and UCP5 (also known as BMCP1) that are more divergent [3]. UCP2 is a member of the mitochondrial anion carrier family and displays 60% sequence identity with the well-known thermogenic UCP1 present in the brown adipose tissue [2]. The genes encoding UCP2 and UCP3 are adjacent in all species, and they map on mouse chromosome 7, rat chromosome 1, and human chromosome 11 [3]. UCP1–UCP3 genes share six coding exons and have two additional nontranslated exons [4]. UCP2 is highly conserved across species with a 95% homology in the amino acid sequence between rat and human [4]. The UCP2 promoter contains putative response elements for peroxisome proliferator-activated receptor (PPAR*γ*, PPAR*α*, and PPAR*δ*), sterol-responsive element-binding protein (SREBP), and cyclic AMP response element-binding protein (CREB) [5–7].

Several regulatory factors of UCP2 act at both transcriptional and posttranscriptional levels. Purine nucleotides have been recognized to negatively regulate UCP2, whereas unsaturated free fatty acids (FFA), glucose, retinoic acid, lipopolysaccharides, and ROS have been reported to activate UCP2 [8, 9]. In particular, FFA have been demonstrated to strongly enhance UCP2 mRNA expression acting on either PPAR or SREBP element [10].

**Figure 1:**
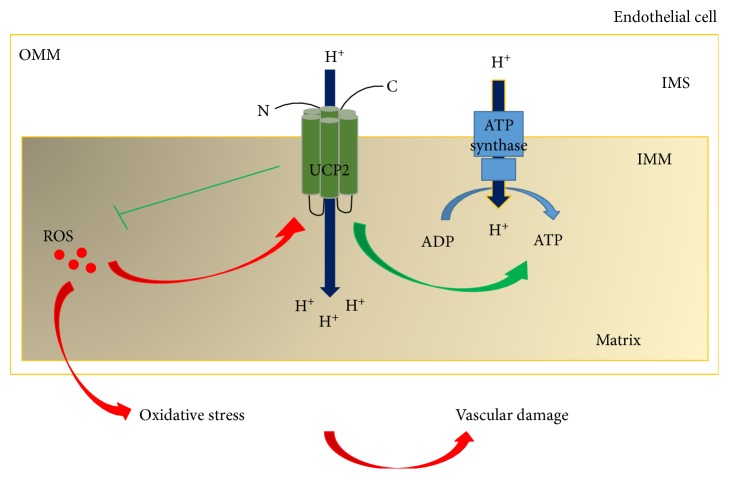
Overview of UCP2 localization and function. UCP2 is located within the inner mitochondrial membrane where it uncouples proton flux with the consequent regulation of ATP synthesis. ROS activates UCP2. The latter, through a negative feedback mechanism, decreases ROS and protects vascular cells from the oxidative stress-dependent damage. See text for details. OMM: outer mitochondrial membrane; IMS: intermembrane space; IMM: inner mitochondrial membrane.

UCP2 is highly expressed in human white adipose tissue. Considerable amounts are also expressed in the skeletal muscle, heart, lung, spleen, thymus, cells of the immune system, and vascular cells, whereas lower amounts are expressed in the brain, liver, and kidney [11]. All members of the UCP family are integral transmembrane proteins, located within the inner mitochondrial membrane. They are characterized by three repeat domains each formed by two *α*-helix regions in the lipid bilayer. The carbossi and aminoterminal regions reside into the mitochondrial intermembrane space whereas the *α*-helix regions are connected by long hydrophilic loops in the matrix side (Figure 1) [12, 13].

UCP2 is involved in the control of uncoupling of proton flux within the mitochondria. In particular, protons generated across the inner mitochondrial membrane during oxidation of substrates reenter in the matrix through UCPs instead of the ATP synthase [14]. Therefore, the proton gradient is converted into heat energy and not in ATP (Figure 1). The involvement of UCP2 in ATP production was demonstrated for the first time in pancreatic *β*-cells [15]. UCP2, unlike UCP1, appears to contribute mainly to the regulation of ATP [16], mitochondrial membrane potential (Δ*ψ*) [17], cellular calcium homeostasis [17], cell survival [18], lipid metabolism [19], and the generation of ROS [19, 20]. UCP2 belongs to the mitochondrial antioxidant mechanisms [21], and as such, it contributes to maintaining the balance between production and clearance of ROS.

Due to the roles that UCP2 plays within the mitochondria, it is clear that a decrease in UCP2 expression may be strongly implicated in the genesis of mitochondrial dysfunction, ROS accumulation, and cell death and, therefore, in the pathogenesis of several diseases.

Whereas controversial evidence exists, within the cardiovascular system, in regard to the role that UCP2 plays in the pathogenesis of left ventricular hypertrophy [22, 23], more robust and convincing findings were reported in relation to UCP2 and its contribution to vascular damage.

The present article will review the available evidence demonstrating the direct contributory role of UCP2, through an increased rate of ROS generation, in the pathogenesis of vascular diseases, particularly within the context of diabetes and hypertension, therefore supporting it as an attractive therapeutic tool for the treatment of the vascular complications associated with these common pathological conditions.

## 2. UCP2 and the Pathogenesis of Vascular Diseases

An increased ROS accumulation is an important mechanism involved in the development of vascular diseases. At physiological concentrations, ROS, produced from both cytoplasmic and mitochondrial sources, represent an intracellular and intercellular second messenger that modulates several downstream signaling molecules leading to vascular reactivity [24], vascular smooth muscle cell growth and migration, expression of proinflammatory mediators, and remodeling of the extracellular matrix [25]. On the other hand, an accumulation of ROS is dangerous. At the level of mitochondria, it leads to damage of mitochondrial proteins, lipids, and DNA with consequent alteration of the mitochondrial function, a further increase in ROS, and, at the vascular level, development of endothelial oxidative stress [26]. In fact, mitochondrial dysfunction causing excessive ROS production contributes to the pathogenesis of several diseases including cardiovascular diseases [27]. It has been demonstrated that UCP2 proton leak is activated directly by superoxide and ROS by-products [9], thus creating a local feedback circuit. Although the mechanism by which UCP2 is activated by ROS is not completely understood, reactive aldehydes derived from lipid peroxidation have been suggested to be the ROS able to induce UCP2 activation [28]. The known intracellular signaling pathways mediating UCP2 gene activation are the AMPK/SIRT1/PPAR*α* axis [29] and the cAMP-dependent protein kinase [30, 31].

Once activated, UCP2 modulates several proinflammatory and proatherogenic signals working in the vasculature and involved in the pathogenesis of diseases. Among them, tumor necrosis factor *α* (TNF*α*) is the most relevant. Thus, the relationship between TNF*α* and UCP2 expression levels appears to be of importance in assessing the risk of vascular damage. It has been reported that TNF*α* directly downregulates adiponectin [32] thus contributing to the development of vascular insulin resistance and to decreased UCP2 levels in the aorta. On the other hand, higher levels of adiponectin might induce UCP2 overexpression in the aorta attenuating the vascular damage [33]. Another mechanism involved in the inhibitory effect of TNF*α* on UCP2 expression is the nitric oxide- (NO-) dependent pathway that involves the inducible nitric oxide synthase (iNOS) expression in both endothelial and vascular smooth muscle cells [34]. Thus, based on the evidence that TNF*α* downregulates UCP2 expression level, a pretreatment with an anti-TNF*α* antibody has been attempted in vivo [33]. TNF*α* and insulin share an antagonistic effect on UCP2 expression level in vascular cells and also in the aorta in vivo. Moderate hyperinsulinemia in response to insulin resistance or lowering of TNF*α* levels within the aorta attenuated vascular damage [33]. This protective effect was mediated by increased UCP2 expression level through a decrease in iNOS expression. Insulin might also reduce NF-*κ*B activation in the aorta, and as a consequence, it may favor UCP2 overexpression and may protect against the vascular damage.

A part from TNF*α*, UCP2 modulates the expression of other inflammatory cytokines within the vascular tissue. In fact, a significant increase in the protein levels of cutaneous T-cell-attracting chemokine (CTACK), chemokine (C-X-C motif) ligand-16 (CXCL16), eotaxin-2, fractalkine, and B-lymphocyte chemoattractant (BLC) was reported in the UCP2 gene-knockout mice [35]. The upregulation of these inflammatory mediators contributed to the ischemic brain damage and to the neuronal death after transient focal ischemia in the UCP2-knockout model. On the other hand, UCP2 overexpression in monocytes led to a significant reduction of *β*_2_ integrin level with a consequent decrease in monocyte adhesion and transendothelial migration [36]. The latter phenomena can contribute to the reduction in atherosclerotic plaque formation in the presence of higher UCP2 level.

UCP2 has also been reported to prevent mitochondrial-induced cell death [18]. Although the precise mechanism is not completely understood, several authors suggested that UCP2, acting on multiple aspects of mitochondrial functions (Δ*ψ*, ATP/ADP ratio, calcium transport, and levels of ROS), may reduce the activation of the mitochondrial permeability transition pore (mPTP), the main activator of mitochondrial death cascade [17].

### 2.1. Atherosclerosis

Atherosclerosis is a disease of the large vessels where it leads to plaque deposition and narrowing of the lumen with distal ischemia. Microvascular disease affects the small blood vessels of highly perfused organs with low vascular resistance such as the retina, kidney, heart, and brain [37]. The pathogenesis of both macro- and microvascular diseases is associated with several risk factors and common pathological conditions such as hypertension, diabetes, obesity, and dyslipidemia [38, 39]. This process involves multiple molecular mechanisms, primarily oxidative stress and inflammation.

Evidence of a direct role of UCP2 in the promotion of atherosclerosis in vivo was first obtained in the UCP2 gene-knockout mice that developed multiple atherosclerotic lesions in several districts, with a significantly shorter lifespan [40, 41]. Moreover, the bone marrow transplantation from UCP2-deficient mice to low-density lipoprotein receptor (LDLR^−/−^) mice markedly increased atherosclerotic lesion size [42]. An indirect evidence of the pathogenic link between UCP2 and vascular damage was provided by the high-fat diet-fed BATIRKO mice that manifested higher oxidative stress in the aorta in association with lower UCP2 expression level [33]. The decrease in UCP2 level in the aorta was strongly inversely correlated with lipid accumulation and lesion area in the normoinsulinemic BATIRKO mice [33].

On the other hand, UCP2 overexpression has been shown to ameliorate both hyperglycemia and obesity-induced endothelial dysfunction [43, 44], and it may help to prevent the development of atherosclerosis in patients with increased ROS, such as those with diabetes, obesity, or hypertension [45].

### 2.2. Diabetes

The pathogenetic link between UCP2 and metabolic diseases, involving the associated vascular damage, is complex. First of all, UCP2 might play an important role in the regulation of energy expenditure and it is likely to contribute itself to obesity and type 2 diabetes mellitus (T2DM). In fact, reduced UCP gene expression was found in tissues of obese subjects and in the kidneys of T2DM patients [46, 47]. Furthermore, few case-control genetic association studies reported significant associations between UCP2 gene polymorphisms and obesity and diabetes in humans [47–52]. On the other hand, both obese and diabetic patients have associated vascular complications such as atherosclerosis [53] and retinopathy [54] where UCP2 may play a direct contributory pathogenetic role. In this regard, the early stages of diabetic retinopathy, the most common complication of diabetes, are characterized by microvascular cell damage [54] associated with the thickening of the capillary endothelial basement membrane and pericyte apoptosis induced by hyperglycemia [55]. Previous investigations into the molecular mechanisms that cause this common vascular complication of diabetes focused on the vascular endothelial growth factor (VEGF) [56, 57]. Recently, the important role played by increased mitochondrial ROS production in the complications of diabetes, including retinopathy, has been emphasized [58]. Of note, UCP2, as a mediator of the mitochondrial ROS pathway, appears to be involved [59].

Recent studies investigated the possible inhibition of high glucose-induced apoptosis by UCP2 in human umbilical vein endothelial cells (HUVEC) [60] and demonstrated that UCP2 plays an antiapoptotic role in this experimental setting. The beneficial effects of UCP2 on the vascular consequences of hyperglycemia are interesting based on the known observation that glucose itself is responsible for the vascular complications and that, as a downstream effector of hyperglycemia, oxidative stress is involved in the development of vascular dysfunction. Glucose can induce ROS generation by multiple mechanisms including the mitochondrial respiratory chain [61]. The endogenous antioxidant system should counterbalance the ROS production in the vasculature, but it fails to do so in diabetes, leading to oxidative stress accumulation and damage [62]. Consequently, the restoration of the mitochondrial antioxidant mechanisms can represent a useful approach for the prevention and treatment of diabetic vascular complications and of the consequent clinical events. The potential beneficial effect of this strategy is supported by evidence that a common −866G>A variant in the promoter of the UCP2 gene, where the A allele associates with increased gene expression [49], decreased the risk of myocardial infarction, angina pectoris, coronary artery bypass grafting (CABG), and sudden death in TDM2 patients [52, 63].

### 2.3. Hypertension

A tight link exists also between UCP2 and vascular damage in hypertensive disease, particularly in the presence of excess salt intake. Abundant evidence in the literature demonstrates that high salt (HS) intake is a major factor increasing systemic blood pressure (BP) levels and thereby vascular damage [64, 65]. HS diet plays also direct harmful effects independent of its impact on BP levels [66]. The mechanisms underlying the direct deleterious effect of HS intake on the vascular bed have not been fully understood. Recent studies demonstrated an important role of UCP2 in the salt sensitivity and its vascular consequences. In this regard, it has been shown that HS diet caused enhanced BP levels and an impairment of vascular function (reduction in endothelium-dependent relaxation) in UCP2^−/−^ mice [67, 68]. On the other hand, overexpression of UCP2 was able to ameliorate the salt-induced vascular dysfunction [69]. The beneficial effect of UCP2 was mediated by decreased superoxide production and increased NO bioavailability. In fact, UCP2 deficiency exacerbated the HS-induced reduction of NO bioavailability and increased superoxide production.

Consistent with the above-mentioned evidence, the gene encoding UCP2 has been underscored as a contributory factor to the HS diet-dependent vascular disease in an animal model of spontaneous hypertension, renal damage, and stroke. In fact, it was found that UCP2 maps nearby the lod score peak of a quantitative trait locus for stroke (STR1) in the HS-fed stroke-prone spontaneously hypertensive rat (SHRSP), a strain with increased susceptibility to vascular damage [70]. Recent studies performed in this animal model, aimed at the further dissection of the pathogenetic contributory role of UCP2 in the vascular disease of the strain, showed that a differential regulation of UCP2 expression, and of all proteins that lie upstream in the UCP2 regulatory pathway, was present in the kidneys of SHRSP, but not in those of the stroke-resistant SHR (SHRSR) upon the stroke-permissive HS dietary regimen. In particular, we obtained evidence of reduced UCP2 gene and protein expression in the kidneys of stroke-prone rats and of increased expression in the kidneys of stroke-resistant rats. Moreover, knocking out the UCP2 gene in mesangial renal cells led to increased oxidative stress, reduced ATP synthesis, and increased cell necrosis [71, 72]. Furthermore, a recent study demonstrated that proximal tubular epithelial cells from the SHRSP strain, once exposed to the high-NaCl medium, showed a significant UCP2 downregulation with an increased rate of cell inflammation and necrosis. Notably, the renal tubular epithelial cells recovered their healthy status after exposure to exogenous recombinant UCP2 despite the presence of high-NaCl medium [73]. These results strongly suggest that changes in UCP2 expression contribute to the higher predisposition of HS-fed SHRSP to renal damage development.

Similar findings were more recently obtained in the brain of the SHRSP, compared to the SHRSR, with evidence of a significant contributory role of reduced UCP2 gene and protein expression in the higher stroke predisposition of the strain [74].

Moreover, a striking downregulation of UCP2 gene and protein expression was found in relation to both hypertension and ageing in the brain, heart, and kidneys of the SHRSP, but not of the SHRSR [74]. In fact, the age- and hypertension-related UCP2 suppression in SHRSP associated with a higher oxidative stress accumulation and inflammation in all tissues examined, along with an increased rate of interstitial, perivascular, peritubular, and glomerular fibrosis in the kidneys; with increased percentage of perivascular and myocardial interstitial collagen accumulation in the heart; and with higher occurrence of ischemic and hemorrhagic lesions in the brain [75]. The latter study supported the concept that, through greater oxygen consumption, reduced mitochondrial membrane potential, and reduced ROS generation, all underlying positive influences on biological ageing, UCP2 may also contribute to expand lifespan, as stated in the “uncoupling-to-survive” hypothesis [76].

Thus, HS diet, high BP levels, and ageing are able to turn off tissue UCP2 gene expression only in the SHRSP animal model through still unexplained mechanisms, therefore contributing to the higher susceptibility to vascular disease of the strain. Interestingly, an epigenetic regulation may be involved in the selective UCP2 gene downregulation upon HS diet in SHRSP, as suggested by the evidence obtained in our studies in relation to both kidney injury [71] and brain damage [74]. Based on the above-reported evidence, an increase in UCP2 expression may play a compensatory role to offset, at least in part, the salt-induced endothelial dysfunction and vascular damage in hypertension with a consequent net reduction of adverse outcomes.

### 2.4. Stroke

Several findings provide evidence that UCP2 is directly involved in the development of cerebral ischemic damage, independent of hypertension. In fact, consistent with its ability to decrease endogenous mitochondrial ROS production and to maintain normal mitochondrial membrane potential and ATP levels, a neuroprotective effect of UCP2 has been described both in vitro and in vivo [18, 35, 77–80]. In this regard, both the enhanced levels of ROS and the apoptosis of neuronal cells are considered important contributory factors to the cerebral ischemia/reperfusion injury (I/R), a condition of great importance among all types of stroke [81]. Of note, it has been reported that sestrin2 (Sesn2), a powerful free radical scavenger, exerts a neuroprotective effect during cerebral I/R injury possibly by increasing mitochondrial biogenesis. Sesn2 is a member of the sestrin family, which is a group of highly conserved proteins that are induced by environmental stresses, including DNA damage, oxidative stress, and hypoxia [82]. Importantly, Sesn2 silencing suppressed AMPK activation, leading to downregulation of UCP2, superoxide dismutase 2 (SOD2), peroxisome proliferator-activated receptor-gamma coactivator (PGC)-1*α*, and the downstream signaling factors with resulting decreased mitochondrial biogenesis and enhanced intracerebral oxidative stress. Thus, Sesn2 provides useful indirect evidence of the beneficial role of UCP2 in stroke.

### 2.5. Peripheral Vascular Disease

Evidence obtained by a microarray approach in human aortic specimens demonstrated that the UCP2 gene is markedly downregulated in the aortic occlusive disease [83], a common cause of morbidity and mortality in elderly population. Moreover, a study performed in a Caucasian general population revealed that the −866G>A variant of the UCP2 promoter was modestly associated with asymptomatic carotid atherosclerosis in middle-aged women [84].

## 3. UCP2 as a Potential Therapeutic Target for the Treatment of Vascular Diseases

Several studies suggested that excessive ROS production is involved in the atherosclerotic plaque formation [85] and that all plaque cellular components may respond to and be damaged by ROS. The latter contribute to plaque progression and finally to plaque rupture [86]. Thus, several approaches to stop ROS production and to modify disease progression may be used to treat atherosclerosis and its dreadful consequences.

Although UCP2 negatively regulates intracellular ROS production and protects vascular function, its contribution to the vascular benefits of drugs used to treat cardiometabolic diseases is not completely known. Both *in vitro* and *in vivo* studies reviewed herein strongly highlight the role of UCP2 as a potential therapeutic target for the treatment of vascular diseases. In particular, UCP2 might function as an adaptive antioxidant defense to protect against the development of atherosclerosis in response to high-fat and cholesterol diets [40, 87] and to improve hyperglycemia-induced endothelial dysfunction [43]. In this regard, diabetes might derive a major benefit from UCP2-targeted therapies. In fact, despite considerable evidence showing beneficial effects of antioxidants, results from large-scale clinical trials led to the conclusion that classic antioxidant scavengers may not be appropriate therapeutics in diabetes [88]. Therapeutic approaches specifically designed to counteract glucose-induced mitochondrial ROS production in the vasculature are expected to show major efficacy against all diabetic complications, but direct pharmacological targeting (scavenging) of mitochondrial oxidants remains challenging due to the high reactivity of some of these oxidant species.

The following paragraphs will discuss the available indirect evidence documenting the role of UCP2 as a mediator of the vascular protective effects exerted by either drugs or vegetable extracts.

### 3.1. UCP2 and Its Involvement in the Effects of Pharmacological Therapy

 It has been recently shown that glucocorticoids induce the expression of the UCP2 protein [89]. In fact, UCP2 silencing prevented the protective effect of glucocorticoids on ROS production. UCP2 induction upon glucocorticoids increased the oxygen consumption and the proton leak in microvascular hyperglycemic endothelial cells [89]. Thus, UCP2 activation may represent an attractive experimental therapeutic intervention for the treatment of diabetic vascular complications. While direct use of glucocorticoids may not be feasible for the treatment of diabetic complications, due to the significant side effects that develop during their chronic administration, the UCP2 pathway may be therapeutically targetable by other glucocorticoid-independent pharmacological tools.

A recent study demonstrated that a highly selective dipeptidyl peptidase-4 inhibitor, sitagliptin, used for the treatment of diabetes, protected endothelial function in hypertension through the upregulation of UCP2 expression and the subsequent scavenging of mitochondrial ROS [90]. The latter phenomenon, in turn, downregulated cyclooxygenase- (COX-) 2 expression via the stimulation of the glucagon-like peptide- (GLP-) 1/GLP-1R/AMPK cascade. UCP2 inhibited oxidative stress and downregulated COX-2 expression through the GLP-1/GLP-1R/AMPK axis. In humans, dipeptidyl peptidase-4 inhibitors appear to have unique and/or additive antiatherosclerotic effects as add-on therapy to statins and/or angiotensin-converting enzyme inhibitors/angiotensin II receptor blockers [91]. Thus, UCP2 is revealed as an effective molecular target for drug intervention to combat both diabetes- and hypertension-related vascular diseases.

In the context of hypertension, the mitochondrial dysfunction contributes to the promotion of target vascular damage [92]. Mitochondrial abnormalities were described in the heart and in the brain of SHR [93, 94], in the kidneys of both renovascular and essential hypertensive animal models [95, 96], and in the brain of SHRSP [97, 98]. Interestingly, a significant protective effect on both renal and cerebrovascular damage was described with fenofibrate in salt-loaded SHRSP [97]. This beneficial effect may be mostly mediated by the peroxisome-proliferator activated receptor alpha (PPAR*α*) agonist action of fibrates with the consequent stimulation, among other properties, of UCP2 expression [99]. The latter may exert protective effects on target organ damage through its action on mitochondrial biogenesis and function. In fact, we recently obtained evidence that fenofibrate stimulates brain UCP2 expression, reduces tissue oxidative stress and inflammation, and exerts full protection from stroke occurrence in salt-loaded SHRSP [74]. At the cellular and tissue levels, PPAR*α* activity mitigates atherosclerosis by blocking macrophage foam cell formation, vascular inflammation, vascular smooth muscle cell proliferation and migration, plaque instability, and thrombogenicity [100]. Interestingly, clinical use of the synthetic PPAR*α* agonist fibrate has been reported to reduce cardiovascular risk particularly in high-risk patients [101].

#### 3.2. The Effects of Vegetables and Plant Extracts on UCP2 Gene and Protein Modulation

 We obtained evidence of a significant stimulation of renal UCP2 expression by the administration of a vegetable extract obtained from *Brassica oleracea* sprouts in the animal model of salt-loaded SHRSP. In fact, the latter was able to counteract the HS diet-induced renal damage of SHRSP, independent of BP levels [102], and to reduce the stroke occurrence of the strain [74]. The key role of UCP2 stimulation in mediating the positive effects of *Brassica oleracea* sprout extract was reinforced by evidence that parallel blockade with a selective inhibitor of PPAR*α* did not allow any further protection from both cerebrovascular and renal damages in HS-fed SHRSP [74, 102]. Therefore, restoring UCP2 expression levels, as observed under the *Brassica oleracea* sprout extract, appears to be a critical step to prevent target organ damage development in hypertension.

Of note, the beneficial cardiovascular effects of vegetable compounds, including extracts from *Brassica oleracea*, were also reported in humans, thus underscoring the potential useful role of this vegetable as an adjuvant to conventional cardiovascular therapies [103].

Interestingly, the improvement of mitochondrial function by the vegetable administration is often mediated through UCP2 upregulation. Curcumin is an extract from the *Curcuma longa* (turmeric) plant rhizome that has been widely used worldwide as a spice and an herbal medicine. Curcumin has many biological activities, including the amelioration of ischemic stroke and antioxidant, anti-inflammatory, and antineurodegenerative properties [104, 105]. Chronic dietary curcumin administration significantly reduced ROS production and improved cerebrovascular endothelium-dependent relaxation in ageing wild-type mice, but not in ageing UCP2^−/−^ mice [106]. Dietary curcumin administration for one month remarkably restored the impaired cerebrovascular endothelium-dependent vasorelaxation in ageing Sprague Dawley rats. In cerebral arteries from this rat model and in cultured endothelial cells, curcumin promoted endothelial nitric oxide synthase (eNOS) and AMPK phosphorylation, upregulated UCP2, and reduced ROS production. The effects of curcumin were abolished by either AMPK or UCP2 inhibition [106]. Thus, the curcumin-mediated UCP2 upregulation involved AMPK activation in the cerebrovascular endothelium. This activation antagonized superoxide anion production and prevented the NO reduction in endothelial cells [106].

Berberine, a botanical alkaloid purified from Rhizoma coptidis and a constituent of many medicinal plant extracts, improves metabolic conditions in dyslipidemia, obesity, and T2DM [107]. Berberine reduces body weight and improves glucose tolerance and insulin action in obese and/or diabetic mice by activating the AMPK [108]. Notably, berberine increases AMPK1*α* and UCP2 mRNA in visceral adipose tissues and livers of high-fat diet-fed mice [109]. In cultured HUVECs, berberine significantly increased UCP2 mRNA and protein expression in an AMPK-dependent manner with promotion of mitochondrial biogenesis [110]. Transfection of HUVECs with nuclear respiratory factor 1- (NRF1-) specific siRNA attenuated berberine-induced expression of UCP2. Through the same mechanism, a significant reduction of atherosclerotic aortic lesions was observed in both ApoE^−/−^ and ApoE^−/−^/AMPK alpha 2^−/−^ mice [110].

Capsaicin is a phytochemical responsible for the spiciness of peppers [111]. It has the potential to modulate the metabolism via activation of one of the components of the transient receptor potential (TRP) channel family, the TRP vanilloid 1 (TRPV1). The TRP channel family plays a key role in the regulation of cellular calcium signaling and of cardiometabolic functions [112]. In particular, activation of TRPV1, found not only on nociceptive sensory neurons but also in a range of other tissues [112, 113], induces calcium influx, and in certain tissues, this is associated with increased activation or expression of key proteins such as eNOS, UCP2, Krüppel-like factor (KLF2), PPAR*δ* and PPAR*γ*, and liver X receptor alpha (LXR*α*) [113]. The increased expression of UCP2 induced by TRPV1 activation exerts a protective antioxidant effect in the liver in the nonalcoholic fatty liver disease and in the vascular endothelium in the context of hyperglycemia. In rodent studies, capsaicin-rich diets have shown favorable effects on atherosclerosis, metabolic syndrome, diabetes, obesity, nonalcoholic fatty liver disease, cardiac hypertrophy, hypertension, and stroke [114]. The upregulation of protein kinase A/UCP2 via TRPV1 activation ameliorated coronary dysfunction and prolonged the lifespan of atherosclerotic mice by improving endothelial mitochondrial function. On the other hand, either TRPV1 or UCP2 deficiency exacerbated high-fat diet-induced coronary dysfunction, and this result was associated with increased ROS generation and reduced NO production [114]. Topical application of capsaicin via patch was found to increase exercise time to ischemic threshold in patients with angina [115]. Thus, dietary capsaicin supplementation may represent a promising intervention for the primary prevention of coronary heart disease [115]. Further clinical studies with capsaicin administered in food and capsules, or via patch, are needed to establish protocols that are tolerable for most patients and to evaluate the potential of capsaicin for promoting vascular and metabolic health.

## 4. Conclusions

**Figure 2:**
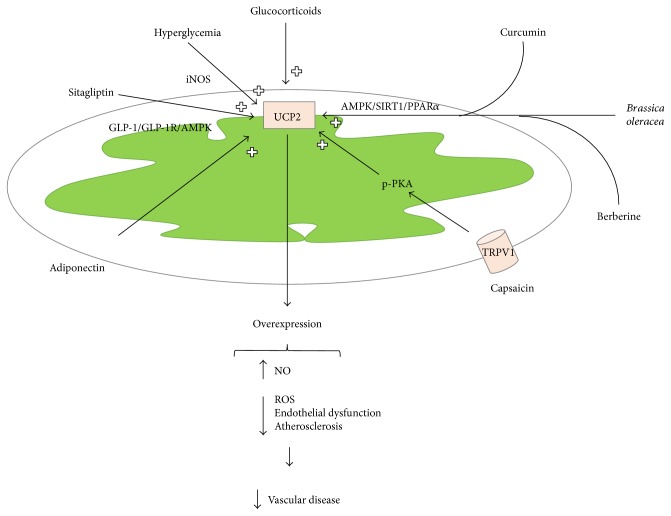
Schematic representation of UCP2 gene upregulation. Schematic representation of the pharmacological, vegetable, and humoral factors known to positively modulate UCP2 gene transcription. As a consequence of the UCP2 protein overexpression, several beneficial effects are observed in the vascular system with a significant reduction of the vascular disease occurrence.

Mitochondrial dysfunction is an emerging contributory mechanism into the pathogenesis of human vascular diseases. For these reasons, novel targeted approaches, designed towards mitochondrial-dependent pathogenic mechanisms, may reveal a useful therapeutic strategy through their ability to improve mitochondrial function and therefore to protect patients from cardiovascular disease occurrence [116]. Among the mitochondrial proteins, UCP2 has been revealed as a significant player in the protection from vascular damage since lack of UCP2 leads to increased micro- and macrovascular damage in distinct pathological contexts. The fine molecular mechanisms dependent on the lack of the UCP2 protein, such as ROS accumulation, upregulation of inflammatory cytokines, increase in monocyte adhesion and transendothelial migration, and increased cell death, may well explain the observed increased rate of atherogenesis and related clinical consequences, as well as the shorter lifespan. Therefore, UCP2 may represent an attractive and promising therapeutic tool in cardiovascular therapeutic. Based on the current knowledge that has been reviewed herein, particularly on the effects of sitagliptin, fibrate, and vegetable extracts, it is likely that overexpression of UCP2, with a consequent improvement of mitochondrial biogenesis and function, may represent a mechanism by which a therapeutic agent can modulate vascular oxidative stress and exert vascular protection (Figure 2). However, the regulation of UCP2 expression is still a complex and incompletely understood process, and there is no specific pharmacological tool available yet to induce UCP2 gene transcription.
